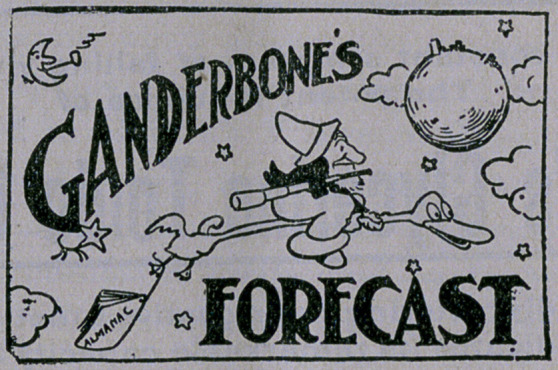# Abstracts and Selections

**Published:** 1908-11

**Authors:** 


### Abstracts and Selections.

**For November.**

(Copyright 1908, by C. H. Rieth.)

In the old Roman calendar November was the ninth month. Blessings fell early, and the empire gave thanks just before the first frost; but about 700 B. C. the trusts left the people so little to be thankful for after nine months that it was decided to wait a while and see if anything would come of the Roman elections. Numa accordingly made November the eleventh month and had Thanksgiving fall with the first snows, notwithstanding the month gets its name from the Latin novem (nine).

The frisky colt will sniff the air and hear the whistling quail, and the festive calf will indicate the zenith with his tail. The frost will paint the forest with a deep and redder dye, the hired man will shuck the corn, the pumpkin vine will pie, the politicians will hit up their office-holding feud, and the modest maple tree will blush and come out in the nude.

And then the presidential race
  Will hold its royal sway,
And everyone will exercise
  His liver, anyway.
He'll bounce it up and down between
  His pancreas and gizzard,
And waltz it through his inner works
  From A around to Izzard,
And even though his present race
  May prove to be in vain,
He'll have the health and strength to run
  Sometime, perhaps, again.

At any rate the candidates will dash into the stretch, and both Bills-o'-the-Wisp will make themselves quite hard to ketch. They'll spurt in spirited response to many wild arousers, and fan the dust up with the slack downhanging from their trousers. They'll come in sight exhibiting a score of fancy paces, and only hit the trembling earth in four or five high places. The air will darken with the flight of gravel, dirts and sods, and the crowd will sound its battle cries and give and offer odds. And Teddy meanwhile will wedge in quite close beside the track, with something that he has concealed within a paper sack, and when his entry charges down, hot-footing like the wind, T. R. will hang a hornet nest upon him down behind.

And then there will be doings on
  This agitated sphere—
And earth will pitch and buck to beat
  A frenzied Texas steer.
The sun will spin around and around
  And blow up once or twice,
The moon will turn a very dark
  And bloody ball of ice,
And no one will remain to see
  Who won the race for vice.

The man who made election bets,
  Relying on his knowledge,
Will write a sad note to his son,
  Withdrawing him from college.
The football season will wind up,

The class room claim its biaves,
And the facutly will order flowers
And decorate the graves.

Mr. Roosevelt's annual proclamation advancing the price of turkey 10 cents a pound will be issued about the middle of the month. He will urge us to give thanks that 55,000,000 cubic feet of earth were excavated in Panama in October.

The sad-faced gobbler will address
His young and tearful flock,
And clip for memory's sweet sake
A small and tear-stained lock,
And then, with many sighs, will lay
His head upon the block.

Until the 25th November will be under the zodiacal sign of Scorpio. People born in Scorpio are cross at supper, and it is better, if possible, to be born after the 25th, when the month is under the sign of Sagitarius the Archer. Sagitarius people are only cross at breakfast, when everybody is.

Along about the 29th
The Duke of the Abruzzi
Will get it all fixed up that he
Shall wed his tootsey-wootscy;
And Elkins pere will dance a jig
And dream of wedding cake,
While everybody else makes bets
On whether it will take.

And then December will blow in with cold and Christmas glee, and old King Coal, the merry old soul, will thunder out, "Pay me!"

---

### A New Dietetic and Injection Method of Treating Typhoid Fever, with Report of 138 Consecutive Cases Successfully Treated in the Last Ten Years.

Under the above title Dr. F. J. W. Maguire, of Detroit, contributes an interesting article to the July, 1908, issue of the *Michigan State Medical Society Journal.* He bases his conclusions upon experience gained in the United States Marine Hospital Service and in private practice. In part, he says: "I

noticed when treating children with summer diarrhea that shortly after giving them nitrogenous food in the form of milk or beef tea their temperature would always rise. I found that by giving these children a carbohydrate diet in the form of barley or rice water I rarely had a rise in temperature. With this observation in mind and remembering the results found in my autopsies following typhoid, I came to the conclusion that milk as a diet in typhoid fever should be eliminated. To further strengthen this theory I determined to carefully watch the results following the use of carbohydrate diet in the form of rice or barley water, etc. In eighteen cases I found the temperature rise following the milk diet, while there was no perceptible increase in temperature after taking rice or barley water.

"I need scarcely add that as a food in typhoid fever I have never since used milk. It is my practice, when I first see a typhoid fever case, to give plenty of sterile water by mouth for five to ten days or until the patient seems to require nourishment, then I use the peptonoids well diluted with sterile water, and the various flavored ices and gelatines. I condemn cow's milk, as it is a culture medium and the cause of a great deal of local irritation."

With reference to treatment, the doctor states: "Having eliminated the milk diet with its terrible irritating effects in the already inflamed Peyer's patches, half the battle is won. This brings us to a consideration of the therapeutic aspect of this subject. In taking up the use of carbolic acid as the therapeutic agent in typhoid fever, I at first thought that I had discovered means whereby I could abort the disease. I commenced by giving half-dram doses of carbolic acid in a pint of sterile water as an enema. This I found very severe. The temperature would drop from 104 to subnormal, and the patient showed signs of carbolic acid poisoning. The temperature would run from normal to 100 for a few hours, then resume its course. The kidneys were carefully watched in all these cases, as they are the filters by which the toxines are eliminated. In my next series of experiments I began with one drop of carbolic acid in a pint of sterile water given as an enema; if the temperature was not reduced I gave another enema in three hours with two drops, and so on increasing until I gave as high as ten drops or the tolerance of my patient allowed. My next series of experiments was with the drop method of injection. I mixed three to five drops of carbolic acid in a pint of sterile water, placed the solution in a fountain syringe alongside the bed and about a foot above the patient, and allowed about one hour for the solution to pass into the rectum. This was regulated by a gauge with a water-glass attachment, which shows how fast the water drops. Through the reverse mucous currents this solution is carried throughout the intestinal tract and through this large area of absorption is carried to every tissue in the body."

In conclusion, the author says: "I do not limit the use of carbolic acid injection to typhoid fever. I have met with phenomenal success with this mode of treatment in reducing temperature in pneumonia and gastritis and have carried cases of acute appendicitis to a subacute or chronic form, thereby lessening the danger from infection at the time of operation. In these 138 cases reported here today the ages ranged from 3 to 78 years. I gave no cold baths, but applied ice bags over abdomen, and one bath a day for cleanliness. Occasionally I gave a little strychnine, quinine and salol as indicated. Since adopting this dietetic and carbolic injection method of treating typhoid fever, I have treated 138 consecutive cases. This covers a period of about ten years. All these cases responded readily to treatment, notwithstanding the fact that many were advanced before treatment was begun. Four cases had had most profuse hemorrhages, all of which subsided when the milk diet was removed. I blieve by this experiment I have made some very valuable therapeutic and dietetic discoveries, and have sufficient confidence in my treatment that I am compiling a work on the subject."

### Good Cause for Suicide.

William Harman, a resident of Titusville, Pa., committed suicide recently from a melancholy conviction that he was his own grandfather. Here is the singular letter that he left: "I married a widow who had a grownup daughter. My father visited our house very often, fell in love with my stepdaughter and married her. So my father became my son-in-law and my stepdaughter my mother, because she was my father's wife. Some time afterwards my wife had a son; he was my father's brother-in-law and my uncle, for he was the brother of my stepmother. My father's wife—i. e., my stepdaughter—had also a son; he was, of course, my brother, and in the meantime my grandchild, for he was the son of my daughter. My wife was my grandmother, because she was my mother's mother. I was my wife's husband and grandchild at the same time. And as the husband of a person's grandmother is his grandfather, I was my own grandfather.—*Albright's Office Practitioner.*

### Mercury in the Treatment of Tuberculosis.

During the past few months there have appeard in American medical journals several articles upon the treatment of tuberculosis with intramuscular injections of mercury. The beneficial effects of mercury in consumption were discovered accidentally by Surgeon Barton Lisle Wright, of the United States Navy. About three years ago, while on duty in a temporary camp for tuberculous sailors established in Pensacola, he had under his care several patients who were both tuberculous and syphilitic. Mercurial treatment, to his surprise, not only benefited their syphilitic, but also their tuberculous lesions. In September, 1907, there appeared in *Dermatologische Zeitschrift* an abstract of an article by Dr. Wolters in which the efficiency of anti-syphilitic medication in tuberculosis was confirmed. The first report of Dr. Wright's experience was published in the April number of the *U. S. Naval Medical Bulletin,* and as the result of his findings he called attention to (1) the immediate improvement of the patient's general condition, as shown by a slowing of the pulse, reduction of temperature and gain in weight; (2) the fact that mercurial treatment will cure an extremely advanced tuberculous ulceration of the larynx and pharynx in a remarkably short time; (3) the fact that the treatment produces a decided improvement of advanced pulmonary lesions, and also has an evident beneficial action on tuberculous glands. Great care was taken to exclude syphilis in these cases. Large, increasing doses of mercury were required and it was found that consumptives were more tolerant of mercury than syphilitics.

The results of treatment in forty cases of tuberculosis were reported in Surgeon Wright's second paper (*U. S. Naval Medical Bulletin,* July, 1908). Of these cases 70 per cent showed improvement. In a third and latest paper (*N. Y. Medical Journal,* August 29, 1908), published just before we are going to press, Dr. Wright reports sixty-five cases under treatment, with 85 per cent of cases showing improvement. He records in detail the histories of twelve consumptive officers, of whom ten were taking the mercury treatment. Nine of these are rapidly improving, while one has failed slightly. This latest report, accompanied by diagrams of lesions before and after treatment, well illustrates the value of the mercurial treatment in tuberculosis. The method employed by Wright at present consists in giving an intramuscular injection every other day until thirty injections have been given. Then there follows a two week's course of potassium iodide, giving gramme 0.64 thrice a day; then one week's rest from medication, after which the course of injections is repeated. This method is giving the most satisfactory results. While climatic and dietetic measures must remain important factors in the treatment of tuberculosis, any drug which will beneficially influence the ravages of the disease will be welcomed by the medical profession. From the reports already published, mercury seems to have a distinct curative effect upon pulmonary tuberculous lesions, and therefore the probability is that it is destined to play an important rôle in the therapeutics of tuberculosis in the future.

So far as the priority of Dr. Wright's method is concerned, we are writing this just before going to press and have not access to the necessary reference works, but we are under the impression that mercury has been used in the treatment of tuberculosis long before Koch told us the true cause of the disease, at a time when there was no clear distinction between tuberculosis and syphilis so far as etiology was concerned.

However this may be, Dr. Wright has undoubtedly rendered a distinct service to medicine by his work along the lines indicated, and it is far from our intention to minimize the value of his researches.

We have repeatedly urged the use of soluble preparations of mercury, of which the biniodide, in the preparation known as cypridol, is the most efficient. By the use of cypridol, which can be given in sufficiently large doses without any unpleasant effects, we can obtain the required mercurialization in applying the mercury treatment of tuberculosis.—*Editorial La Tribune Medicale,* American Edition, September, 1908.

---

### Tuberculosis Epitomized in a Thousand Words.

BY CARY H. WILKINSON, M. D., Galveston, Texas.

1. Tuberculosis is an infectious disease produced by a germ known as the bacillus tuberculosis, or Koch's bacillus, named after its discoverer, which usually gains access to the human system through the act of respiration, through abraded surfaces on the integument, or through the ingestion of infected milk. It is one of the most fatal diseases known and annually destroys a hundred thousand human beings in the United States.

2. The most common method of conveyance of this germ into the system is through the medium of dried sputum, laden with bacilli, floating about in the air and finding lodgment in the lungs of some uninfected person.

3. This germ rapidly multiplies when once it has gained access to the system, and the rapidity of such development is influenced by the susceptibility of the individual to tuberculosis and by the conditions that surround him after once he has become affected.

4. Many individuals with a strong resisting vitality often escape an outbreak of tuberculosis after once becoming infected, by reason of such vitality, and by observing a proper hygienic existence afterwards, but, on the other hand, all those of feeble resistance, and especially those who lead unsanitary lives afterwards become rapidly contaminated and eventually perish from the effects of this disease.

5. Conditions favorable to the development of tuberculosis in the human system, therefore, are hereditary predisposition, dark, crowded and unventilated dwelling apartments, humidity of the surrounding atmosphere, depressing mental and physical influences and the lack of proper sustenance.

6. On the other hand, conditions opposed to the development of tuberculosis are a strong inherited resisting vitality, a constant supply of pure, fresh atmosphere, an abundance of sunshine, a dry and equable atmosphere, an abundance of nutritious and easily digested food, a purely sanitary environment and the avoidance of all immoderate or debilitating exercises.

7. The bacillus tuberculosis can not thrive in pure oxygen, nor in abundant sunshine, but rapidly multiplies under conditions where these agencies are greatly deficient. Tuberculosis is a disease of crowded life and rarely ever originates and thrives in agricultural districts.

8. In case of infection a person should be sent without delay to some high, dry and salubrious locality, where he should remain permanently, or until entirely cured if possible. Or else he should be sent to some tubercular sanatorium.

9. The premature return of a tubercular person to the locality where the disease originated often means the speedy demise of such individual from tuberculosis. It is, therefore, highly important to recognize the disease as soon as possible. Send the invalid away without delay and keep him in some healthy locality as long as practicable.

10. There are three methods by which tuberculosis may be detected in its incipiency. One is through the agency of the microscope; another through injection of tuberculin into the system; and third, by instilling tuberculin between the eyelids. The latter is known as the ephthalimic test, and is the one to be preferred.

If impractical to send a tubercular case to a sanatorium or to a suitable climate, then observe, as far as possible, the following rules for his hygienic treatment at home.

1. Place such tubercular person in a large, well-lit and well-ventilated room, and keep him there alone during the night time.

2. A tubercular patient's room should be high above the ground and located on the south side of the dwelling. It should embrace an area of at least 4000 square feet, be furnished with openings on every side, if possible, and should be devoid of all interior furnishings, such as carpets, matting, curtains, cushions, soft chairs and surplus furniture. It should not be papered, but either ceiled or plastered.

3. Such room should be kept scrupulously clean all the time. Disinfect once a week by mopping off all exposed surfaces with Kreso, and keep all doors and windows open day and night, except in wet and windy weather the windward openings only should be closed.

4. The bed of a tubercular patient should be placed between two openings in the room so that fresh air may reach him all the time. There should be no canopy, bar or netting to the bed, and his pillows should be encased in oiled silk coverings over which clean linen pillow slips should be worn all the time.

5. A porcelain urinal should be placed alongside the bed, into which the patient should expectorate, and this urinal should be emptied, cleaned and disinfected every day.

6. Tubercular cases should be provided with their own toilet service, towels, brushes, handkerchiefs, etc., as well as with their individual table service, as napkins, cups, saucers, pitchers, spoons and glasses, and these should be kept neat and clean at all times and apart from the others.

7. Tubercular men should be kept closely shaven and women should never wear long hair.

8. The diet for a tubercular person should be nutritious and digestible and the opposite variety should be avoided. Fresh milk, eggs, beef, well-cooked cereals, thick broths and soups and vegetable purees should form the basis of the menu. Ardent spirits are deleterious as a rule, but high graded beer in moderation and taken regularly is advisable.

9. Tubercular cases should keep in motion during much of the day time, but violent exercise is very injurious. Vehicle riding is by far the best form of exercise to be taken. At all times walk erect; at night lay straight in bed, and often during the day time take several deep respirations at a time.

10. Tubercular persons should avoid matrimony, nor should they kiss and fondle little children. They should likewise avoid intemperance and excesses of every description.

11. When expectorating they should be sure to deposit their sputum where it can be destroyed. They should never spit upon the ground or floor, but in a pocket cuspidor provided for such purpose, lest the sputum becoming dry should impregnate the atmosphere and thereby infect the healthy.

12. Should cough become annoying, as it may, avoid the use of opiates as much as possible and inhale the fumes of creosote instead, while occupying the room.—*New Albany, Ind., Medical Herald.*

## Figures and Tables

**Figure f1:**